# Skin transcriptome reveals the dynamic changes in the Wnt pathway during integument morphogenesis of chick embryos

**DOI:** 10.1371/journal.pone.0190933

**Published:** 2018-01-19

**Authors:** Husile Gong, Hong Wang, YueXing Wang, Xue Bai, Bin Liu, JinFeng He, JiangHong Wu, WangMei Qi, WenGuang Zhang

**Affiliations:** 1 College of Animal Science, Inner Mongolia Agricultural University, Hohhot, China; 2 Animal Husbandry Institute, Inner Mongolia Academy of Agricultural & Animal Husbandry Sciences, Hohhot, China; 3 College of Veterinary Medicine, Inner Mongolia Agricultural University, Hohhot, China; 4 Inner Mongolia Prataculture Research Center, Chinese Academy of Science, Hohhot, China; Laboratoire de Biologie du Développement de Villefranche-sur-Mer, FRANCE

## Abstract

Avian species have a unique integument covered with feathers. Skin morphogenesis is a successive and complex process. To date, most studies have focused on a single developmental point or stage. Fewer studies have focused on whole transcriptomes based on the time-course of embryo integument development. To analyze the global changes in gene expression profiles, we sequenced the transcriptome of chicken embryo skin samples from day 6 to day 21 of incubation and identified 5830 differentially expressed genes (DEGs). Hierarchical clustering showed that E6 to E14 is the critical period of feather follicle morphogenesis. According to Kyoto Encyclopedia of Genes and Genomes (KEGG) pathway analysis of the DEGs, two kinds of Wnt signaling pathways (a canonical pathway and a non-canonical pathway) changed during feather follicle and feather morphogenesis. The gene expression level of inhibitors and ligands related to the Wnt signaling pathway varied significantly during embryonic development. The results revealed a staggered phase relationship between the canonical pathway and the non-canonical pathway from E9 to E14. These analyses shed new light on the gene regulatory mechanism and provided fundamental data related to integument morphogenesis of chickens.

## Introduction

Chicken skin and its appendages are widely studied to understand embryonic organogenesis and biological pattern formation because of their good accessibility. Feather morphogenesis results from a coordinated series of epithelial-mesenchymal signals [1, 2], which initiate epithelial downgrowths to form mature feathers before chicken hatching [3, 4]. The process of feather morphogenesis in particular areas during embryonic development can be divided into the following three phases: micro-patterning, intra-bud morphogenesis, and follicle morphogenesis [5](macro-patterning is the phase of forming these different feather tracts on the body surface of chickens, which was not considered in this study). Each of these steps requires a cascade reaction of molecular signaling pathways.

Evidence from animal studies has suggested that many molecules and signaling pathways play a vital role in hair follicle morphogenesis [6], including Shh [7], follistatin [8], noggin [9], FGF2 [10], the BMP pathway [11], the Notch pathway [12], and the Wnt pathway [13, 14]. Numerous studies have shown that Wnt (originally named for the *Drosophila* wingless (wg) phenotype) signaling pathways are essential for the morphogenesis of hair follicles. The Wnt pathway, along with its downstream effector β-catenin, plays an important role in cell proliferation, epithelial architecture, and cell polarity regulation. Two Wnt signaling pathways have been characterized: the canonical Wnt pathway (the Wnt/β-catenin pathway) and the noncanonical Wnt pathway (including the Wnt/planar cell polarity pathway and the Wnt/calcium pathway) [15].

Previous studies focused only on single genes or several gene expression patterns during chicken embryo development. According to a previous report, there is limited information regarding the global genetic basis underpinning the development phase of feather morphogenesis [5]. Next generation sequencing technologies and systems biology provide a new way to define gene expression profiles associated with different stages of an organism’s development.

In this study, we collected the back skin of chickens on days 6 (first record of feather germ development [16]) to 21 of incubation. Transcriptome analysis was then performed to identify genes associated with feather morphogenesis. The result of hierarchical clustering analysis of the expression profiles showed that the phase from E6–E21 was divided into two large stages: feather follicle morphogenesis (E6–E14) and feather morphogenesis (E15–E21). Subdivision of these large stages could provide a new avenue to understand the process of feather morphogenesis. Furthermore, the results showed that genes encoding proteins of the canonical and non-canonical Wnt signaling pathways have different expression patterns during skin morphogenesis. We believe that the results of this study will revive the interest of research groups in the fundamental process of feather morphogenesis.

## Material and methods

### Animal ethics statement

Chicken embryos were killed by cervical dislocation. All animal experiments were performed in accordance with the “Guidelines for Experimental Animals” of the Ministry of Science and Technology (Beijing, China). The experimental procedure was approved by the Animal Care and Use Committee of Inner Mongolia Agricultural University, China.

### Animals and sampling processing

Fertilized chicken eggs were collected from Roman laying hens originating from the College of Animal Science of Inner Mongolia Agricultural University (Hohhot, China). The fertilized eggs were incubated in egg incubators. The hatching conditions comprised a constant temperature and humidity (temperature, 37.5°C; humidity, 60%). The cross region (about 1.5cm^2^) of the midline and two wings of chicken back skin were sampled from E6 to E21. We took 4–5 rows of feathers and with 8–9 feathers per row from one side of the chicken embryo near the midline for transcriptome analysis and 4–5 rows of feathers and with 8–9 feathers per row for histological analysis from the adjacent, contralateral side. The skin part to be used for RNA extraction was preserved in liquid nitrogen immediately. The other part, for tissue sectioning, was preserved in 10% neutral buffered formalin for 20–24 h.

### Total RNA isolation and RNA-seq

Total RNA was isolated from chicken embryo back skin using the TRIzol reagent (Takara, Dalian, China). At each time point, we selected three chicken embryos for skin sampling. We mixed three total RNA samples representing the RNA of each point to construct the transcriptome library. The RNA-seq library was constructed with 150bp paired-end sequences for each sample, according to the standard protocol provided by Illumina, Inc. (San Diego, CA, USA). FastQC was used to calculate the quality control statistics for the data generated by the Illumina HiSeq4000, and the resulting libraries were then subjected to paired-end sequencing (2 × 150bp).

### Transcriptome assembly and differentially expressed genes (DEGs) clustering

We analyzed time-course transcriptomes of dorsal skin during chick embryo development from E6 to E21 (day of incubation). We deposited the transcriptome data in the NCBI database (Accession numbers SRR5922808- SRR5922823) (https://www.ncbi.nlm.nih.gov/bioproject/397795). All clean data were mapped to the chicken genome using TopHat2, with no discordant and mixed parameters. Reference-guided transcriptome assembly, which compensates for incompletely assembled transcripts, was performed using Cufflinks [17, 18] with a bias correction for each sample. The data were then merged into a single unified transcript catalog using Cuffmerge. The comparative transcriptome studies were conducted for 16 samples from the skins of E6–E21 chicken embryos. In this study, 5830 DEGs were identified using EdgeR [19]. Eigengenes were calculated for each gene co-expression module in order to visualize the gene expression patterns for each module using weighted gene co-expression network analysis (WGCNA) [20]. Gene Cluster 3.0 was also utilized to group the DEGs as different expression patterns during the whole developmental process [21]. The R package component, pvclust, was utilized to assess the uncertainty in the hierarchical clustering analysis on 10,000 bootstrap replicates [22].

### Functional annotation and pathway analysis

Functional annotations and pathway analyses of DEGs were carried out using the ClueGO plug-in of Cytoscape [23]. Gene Ontology (GO) annotates genes to biological/cellular/molecular terms in a hierarchically structured manner, whereas the Kyoto Encyclopedia of Genes and Genomes (KEGG) [24] annotates them to functional pathways.

### Histological processing

The skin samples for tissue sectioning were washed with water for more than 8 h. To dehydrate the samples and make them transparent, they were subjected to the following treatment: 50% alcohol for 30 min, 70% alcohol for 30 min, 80% alcohol for 30 min, 90% alcohol for 30 min, 95% alcohol for 30 min, 100% alcohol for 30 min (3 times), 100% alcohol:xylol = 1:1 for 10 min, xylol for 20 min, and xylol for 30min. The formalin-fixed tissues were paraffin-embedded. Serial longitudinal and transverse sections of skin were cut at the desired thickness of 5 μm using a Leica RM2135 microtome (Leica Microsystems, Wetzlar, Germany). After the sections were mounted on slides, the sections were subjected to hematoxylin and eosin staining.

### Validation of RNA-seq data

To confirm the differential expression of genes revealed by RNA-Seq, 11 DEGs were selected for quantitative polymerase chain reaction (qPCR) validation. Primer information is shown in S1 File. Total RNA was extracted using the TRIzol reagent (Takara, Dalian, China) and then converted to cDNA using a PrimeScript RT reagent Kit with gDNA Eraser (Takara) according to the manufacturer's protocol. qPCR was performed on an CFX96 Touch Real-time PCR System (BIO-RAD, California, USA) using SYBR*Premix Ex Taq* II (Tli RNaseH Plus) (Takara). Three technical replicates were performed. The thermal cycling conditions used in the qPCR were 95°C for 5 min; followed by 40 cycles of 95°C for 20 s, the melting temperature of the primers (Tm) for 20 s, and 72°C for 20 s. Relative quantification analysis was performed using the comparative cycle threshold CT method, and the relative gene expression was calculated by the 2^-ΔΔCt^ method [25]. The β-actin gene was used as the reference control.

## Results

### Histological analysis showed that chicken embryo skin morphogenesis occurs through three different processes

The examination of histological sections from E6 to E21 showed that skin morphogenesis during embryonic development could be divided into three processes: Micro-patterning (E6–E8), intra-bud morphogenesis (E9–E10), and follicle morphogenesis (After E11), which were similar to the results of previous studies.

**Fig 1:**
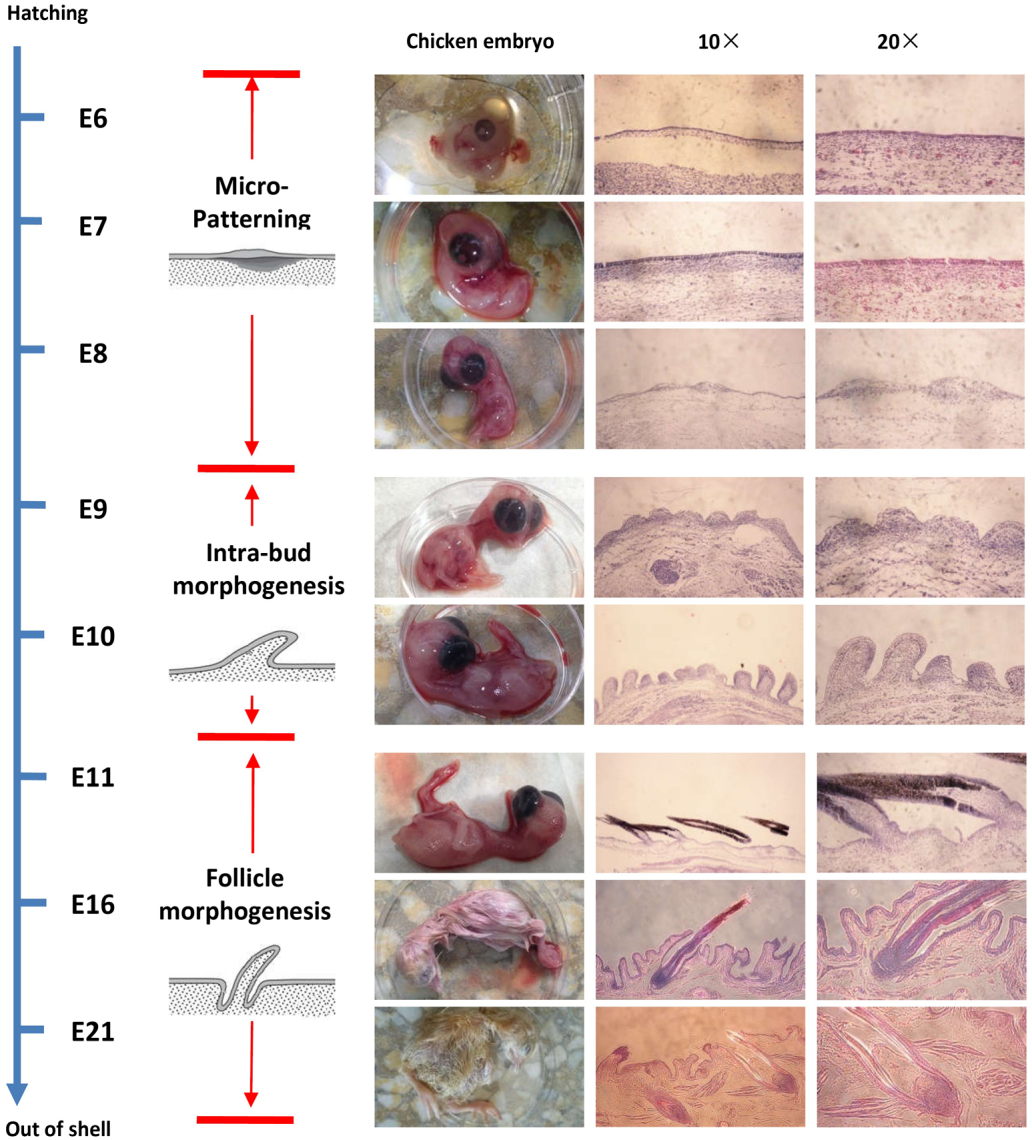
Three different processes in chicken embryo skin development based on morphogenesis. Three different processes in chicken embryo skin development were analyzed: Micro-patterning (E6–E8), intra-bud morphogenesis (E9–E10) and follicle morphogenesis (After E11). Histological sections of three stages of chicken skin during embryonic development (first column: photograph; the second column: Magnified 10×; the third column: Magnified 20×).

During development from E6 to E7, chicken embryonic skin was underdeveloped, and the epidermis layer was very thin and difficult to distinguish among the structures (Fig 1). By E8, an epidermal placode appeared above a condensation of dermal cells that specifies the location of the feather follicle, but did not form the feather buds (Fig 1). When the tract fields are formed, a morphogenesis wave goes through the body surface, leading to the previous fields being changed into buds, which are separated by interbud regions [26, 27]. Therefore, the period from E6 to E8 was categorized as the stage of micro-patterning (Fig 1) [28].

Subsequent proliferation of dermal cells induced by the epidermal placode produced a finger-like feather bud. At E9, short buds were clearly distinguishable in the feather tracts, appearing as symmetrical structures (Fig 1). The buds on the back tract elongated at E10 (Fig 1). During this period, the anterior-posterior axis was forming and the direction of the feathers was established. The period from E9 to E10 was categorized into the stage of intra-bud morphogenesis (Fig 1). As such, the skin transforms from a bud to a feather follicular structure.

The skin began to invaginate to form feather follicles that were prototypes of primary follicles at E11 (Fig 1). The period after E11 was categorized as the stage of feather follicle morphogenesis (Fig 1). As invagination and distal growth continued, the follicles were shaped into a deep, narrow pit, and the feather germs resemble a long cylinder sticking out of the follicles (Fig 1). By E16, feather follicle morphogenesis was mainly completed. Feather fibers continued to elongate, and then differentiated and maturated into the feather follicles (Fig 1).

### The gene expression patterns in skin during chicken embryo development

To comprehensively examine the role of gene expression in the skin during chicken embryo development, we collected the chicken embryo back skin at 16 continuous time points (from E6 to E21) for transcriptome analysis. The analysis results showed that a total of 5830 genes were significantly differentially expressed in at least one of the 16 samples, based on the fragments per kilobase of transcript per million mapped reads (FPKM) value.

**Fig 2:**
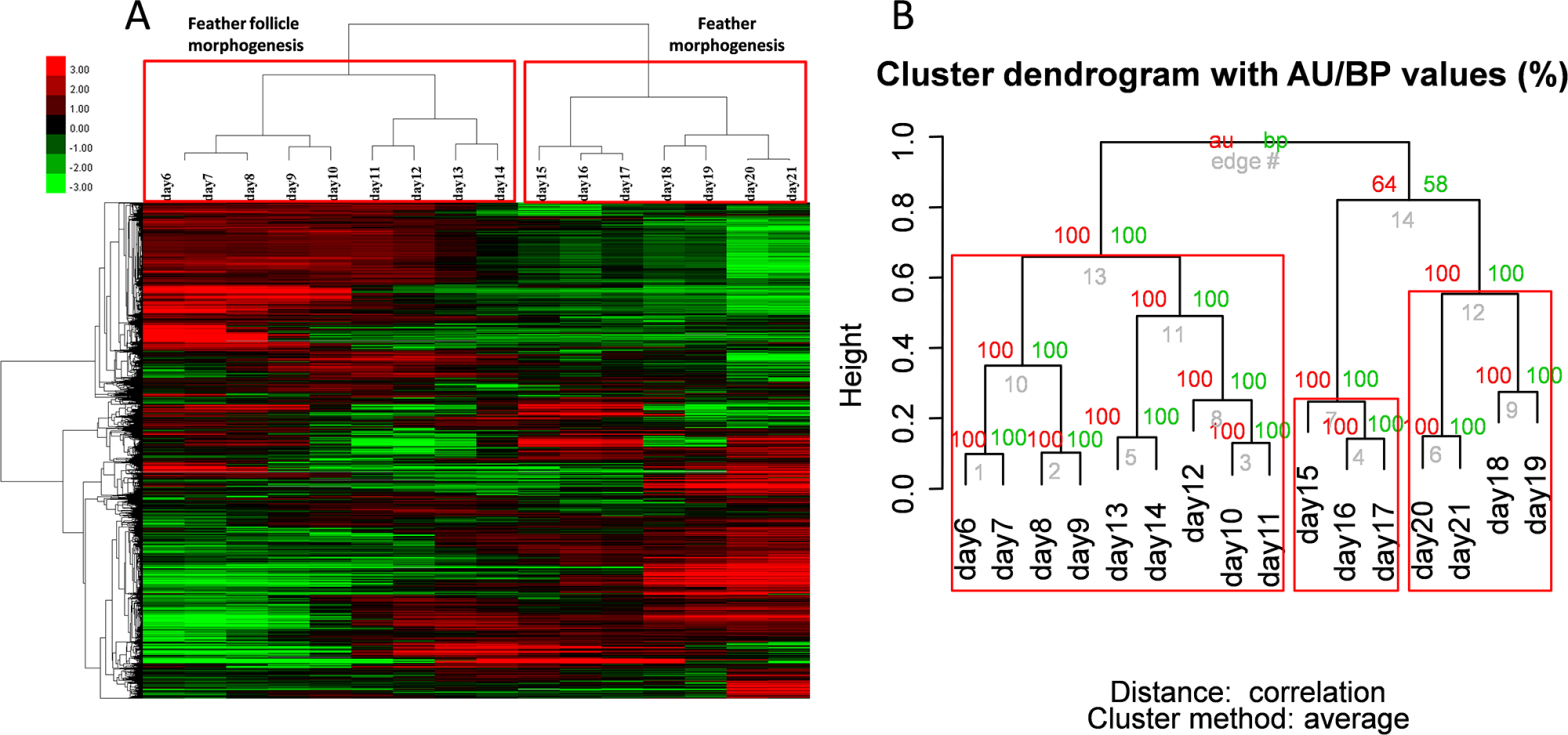
The gene expression pattern in skin during chicken embryo development. (A) Hierarchical clustering analysis of differentially expressed genes. Upregulated genes are displayed as red, whereas downregulated genes are displayed as green. Abscissa: days, ordinate: genes. (B) Hierarchical clustering of 16 time-course transcriptomes performed by pvclust. Values at the branches are Approximately Unbiased(AU) p-values (left), Bootstrap Probability(BP) values (right), and cluster labels (bottom). Clusters with AU ≥ 95 are indicated by rectangles.

Next, we performed hierarchical clustering of the differentially expressed genes (DEGs) according to their expression patterns using Gene Cluster 3.0 (Fig 2A). We the used pvclust to assess the robustness of the hierarchical clustering; except for day 10, the results of pvclust mostly supported those of Gene Cluster (Fig 2B). In the clustering tree, the 16 skin transcriptomes could be divided into two large stages: feather follicle morphogenesis (E6–E14) and feather morphogenesis (E15–E21). We then selected the highly expressed genes in the four subdivisions: E6–E10, E11–E14, E15–E17, and E18–E21 to carry out KEGG pathway analysis (Table 1). Those genes that related to cell adhesion molecules (CAMs), focal adhesion, tight junction, and adherens junction were highly expressed during E6–E10. Those genes that were highly expressed during E10–E14 were associated with the hedgehog signaling pathway and melanogenesis. There were no genes that were highly expressed in skin during E15–E17, which might be because those genes that were highly expressed during E15–E17 were also highly expressed at other stages. We identified 1058 genes as highly expressed during E18–E21, which were mainly involved in fundamental metabolism pathways.

**Table 1:** KEGG pathway analysis clustering of highly expressed for four subdivisions.

Cluster name	the number of genes	KEGG pathway	Associated Genes Found
Cluster(E6-E10)	414	Cell adhesion molecules (CAMs)	CADM1, CDH2, CNTN2, JAM3, NCAM1, VCAN
		Focal adhesion	ACTG1, CDC42, COL9A1, COL9A3, FN1, FYN, GRB2, THBS4
		Pyruvate metabolism	GRHPR, LDHB, PC
		PPAR signaling pathway	FABP7, FADS2, SCD5, SCP2
		Tight junction	ACTG1, CDC42, CTNNA2, EPB41L3, JAM3, MYH15
		Cysteine and methionine metabolism	DNMT3A, DNMT3B, LDHB
		Salmonella infection	ACTG1, CDC42, PFN2, RP11-73M18.2
		Adherens junction	ACTG1, CDC42, CTNNA2, FYN
		ECM-receptor interaction	COL9A1, COL9A3, FN1, THBS4
Cluster(E11-E14)	66	Hedgehog signaling pathway	CSNK1A1, PTCH2, SHH, WNT16
		Melanogenesis	FZD10, KIT, KITLG, TYR, WNT16
Cluster(E15-E17)	0	null	null
Cluster(E18-E21)	1058	Fatty acid degradation	ACSL1, ACSL5, ACSL6, ALDH3A2, CPT1A, HADH, HADHA
		Glycerolipid metabolism	AGPAT1L, AGPAT2, AKR1B10, ALDH3A2, DGAT2, LPIN1, MOGAT1, PNLIPRP3, PNPLA2, PPAP2C
		PPAR signaling pathway	ACSL1, ACSL5, ACSL6, ADIPOQ, CD36, CPT1A, DBI, FABP5, LOC101747587, PLIN1, PPARA, SORBS1
		Peroxisome	ABCD3, ACSL1, ACSL5, ACSL6, HACL1, HSD17B4, PAOX, PEX11G, PHYH, PMVK, SLC25A17
		Focal adhesion	ACTN1, CAV2, CCND1, CHAD, COL1A2, COL4A5, COL6A3, COMP, ITGA3, ITGA8, ITGB4, ITGB5, LAMA3, LAMA5, LAMB3, LAMC1, LAMC2, PDGFA, PDGFB, RAC3, ZYX
		ECM-receptor interaction	CD36, CD44, CHAD, COL1A2, COL4A5, COL6A3, COMP, ITGA3, ITGA8, ITGB4, ITGB5, LAMA3, LAMA5, LAMB3, LAMC1, LAMC2, SDC4

This division has obvious differences from the traditional developmental stages based solely on feather morphology. Combined with the histological results, we found that the period of E6–E14 is an essential stage for feather follicle morphogenesis. To validate our hypothesis, the R package WGCNA [19] was used to analyze the subdivisions for gene expression based on the 5830 DEGs (See S2 File). Among them, five clusters (Cluster day6, Cluster day8, Cluster day10, Cluster day12, and Cluster day14) were associated with the development of the feather follicle. Therefore, we analyzed the relationship between these five clusters and the three morphological stages. We speculated that a cascade effect influenced the level of molecular expression during feather follicle morphogenesis.

### Genes expressed in the skin during micro-patterning

**Fig 3:**
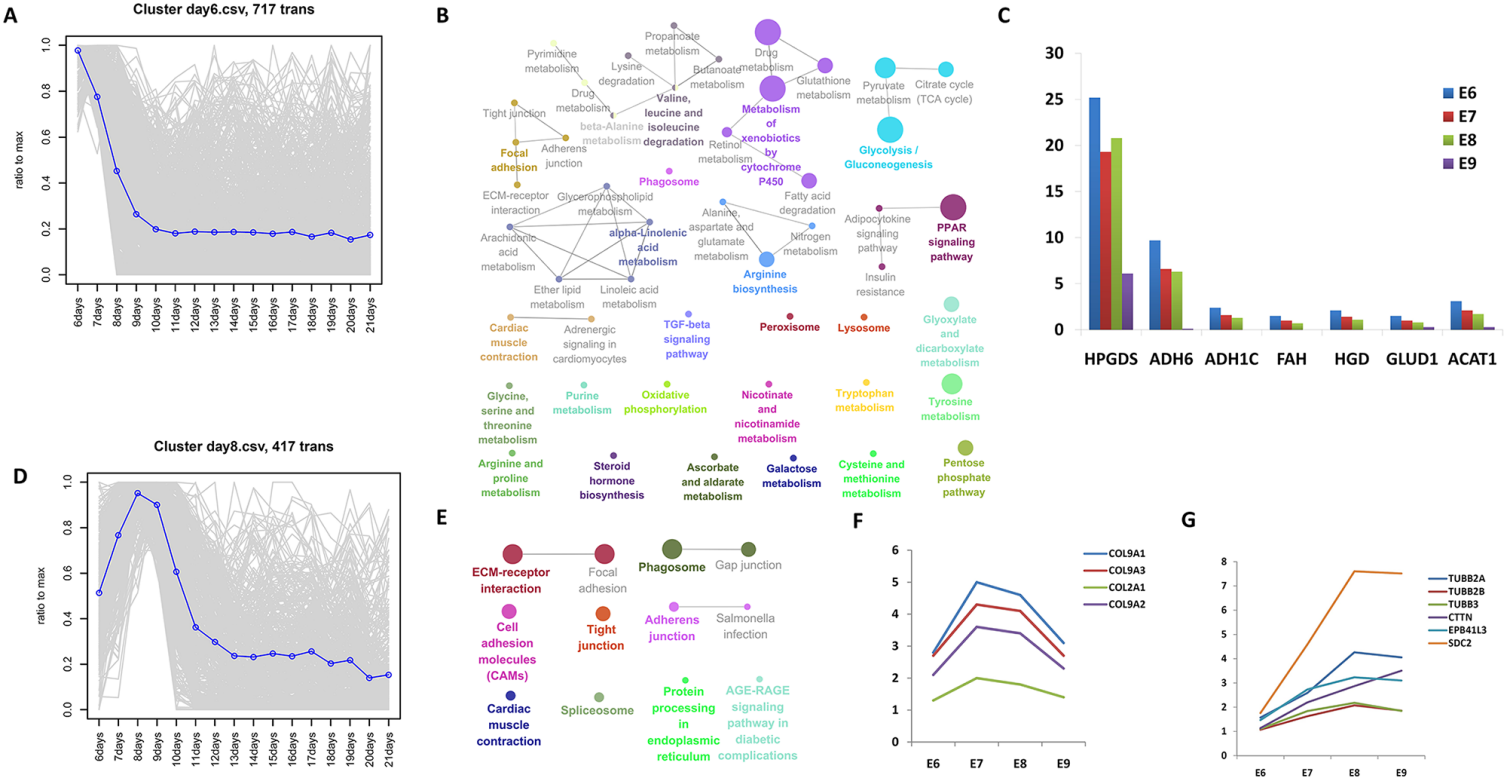
During micro-patterning, the skin expressed genes related to the rearrangement of the cytoskeleton. (A) Cluster day 6 displayed a skin development pattern in micro-patterning (E6–E8), showing a sharp decline in expression from E6. (B) Kyoto Encyclopedia of Genes and Genomes (KEGG) analysis showing the global gene expression patterns from Cluster day 6. (C) The expression of these differentially expressed genes (DEGs) encode core regulatory transcription factors for basal metabolism and amino acid metabolism, declined during the period of micro-patterning (E6–E8). (D) Cluster day 8 displayed skin development patterns that increased sharply during the period of micro-patterning (E6–E8) and peaked at E8. (E) KEGG analysis showing the global gene expression patterns from Cluster day 8. (F) Expression of members of the collagen family began at E6 but was downregulated from E7. (G) Expression of genes associated with the rearrangement of the cytoskeleton began at E6 and continued to increase over the course of time (E6–E8).

Cluster day 6 (717 transcripts) showed a peak at E6 and declined during the period of micro-patterning (E6–E8) (Fig 3A). Pathway analysis showed that genes of core regulatory transcription factors for basal metabolism and amino acid metabolism (Fig 3B), such as *ADH1C* and *ADH6* (glycolysis/gluconeogenesis), *HPGDS* (metabolism of xenobiotics by cytochrome P450), *HGD* and *FAH* (tyrosine catabolism), *GLUD1* (arginine biosynthesis), and *ACAT1* (tryptophan metabolism), were highly expressed at E6 and dramatically downregulated at E9 (Fig 3C).

Cluster day 8 (417 transcripts) showed the greatest increase during micro-patterning (E6–E8) and declined from E9 (Fig 3D). Pathway analysis showed that the DEGs in Cluster day 8 are involved in extracellular membrane (ECM)-receptor interaction, focal adhesion, gap junctions, tight junctions, cell adhesion molecules, and phagosomes (Fig 3E). Collagen (ECM-receptor interaction), the main component of connective tissue, is the most abundant protein in animals. We identified that members of the collagen family (including *COL9A1*, *COL9A3*, *COL2A1*, and *COL9A2*) are expressed in skin at E6 (Fig 3F). Expression of genes related to the rearrangement of the cytoskeleton, *TUBB2A*, *TUBB2B*, *TUBB3* (phagosome), *CTTN*, *EPB41L3* (tight junction), *SDC2* (cell adhesion molecules), started at E6 and continued to increase over time (Fig 3G). Thus, the temporal gene expression pattern might correspond to skin development of the chicken embryo. Interestingly, the expression of *SDC2* was significantly upregulated by 4.5-fold at E8 compared with E6 (Fig 3G). SDC2 plays an essential role in cell binding, cell signaling, cytoskeletal organization, and is related to the Wnt signaling pathways: β-catenin-independent Wnt/PCP signaling pathways. *CDC42*, a gene encoding a protein regulating cell adhesion, migration, and invasion, was upregulated. Moreover, genes related to neural development (*RTN1*, *TAC1*, *VSNL1*, *CDK5R2*, *SCG3*) were significantly upregulated by 16.38-, 16.02-, 5.50-, 3.57-, 3.50-fold, respectively, during micro-patterning (E6–E8). These results suggested that the development of feather follicles and the nervous system might be synchronous at the stage of skin development.

### Dynamic changes in the expression of Wnt signaling pathways during intra-bud morphogenesis (E9-E10)

**Fig 4:**
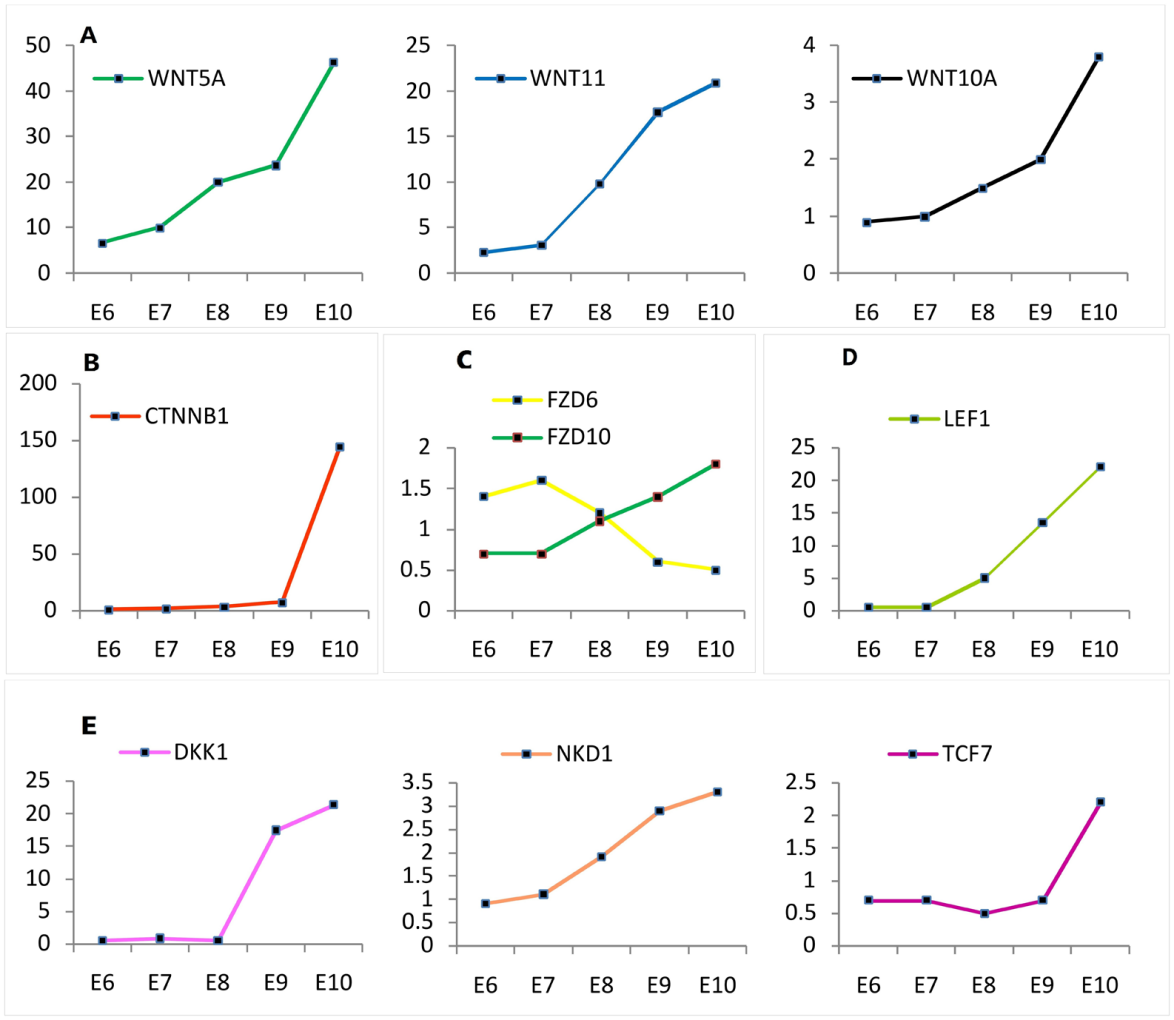
Dynamics of Wnt signaling pathways in intra-bud morphogenesis (E9–E10) during the development of skin in chicken embryos. (A) Expression levels of *Wnt* family members (*WNT5A*, *WNT11*, *WNT10A*) were upregulated at E10 compared with E6. (B) *CTNNB1* expression was dramatically increased by 20-fold during E9–E10. (C) The expression levels of *FZD10* and *FZD6*, encoding the receptors for Wnt proteins, were significantly upregulated and downregulated, by 2.4- and 2.3-fold, respectively, over the course of time. (D) The expression of *LEF*, which encodes a transcription factor involved in the Wnt signaling pathway, was increased by 44-fold at E10 compared with E6. (E) Several genes encoding negative regulators of the Wnt/beta-catenin canonical signaling pathway, such as *DKK1*, *NKD1*, *TCF1*, were consistently upregulated during intra-bud morphogenesis (E9–E10).

During intra-bud morphogenesis, the anterior-posterior axis and direction of feathers are established. The results of WGCNA showed that 173 genes of Cluster day10 were highly expressed at E10 (See S2 File). We identified that the expression levels of members of Wnt signaling pathway changed drastically during intra-bud morphogenesis. Wnt signaling is involved in the control of the molecular and morphological asymmetry of the follicle, and the associated hair shaft. *WNT5A*, *WNT11*, and *WNT10A* were upregulated by 6.7-, 8.9-, and 3.9-fold respectively at E10 compared with E6 (Fig 4A). The expression level of *CTNNB1*, which encodes a protein that acts as an intracellular signal transducer in the Wnt signaling pathway, was dramatically increased by 20-fold from E9–E10 (Fig 4B). In addition, FZD10 and FZD6 are the receptors for Wnt proteins and couple to the Wnt/β-catenin canonical signaling pathway. We found the expression levels of *FZD10* and *FZD6* were significantly upregulated by 2.4-fold and down-regulated by 2.3-fold, respectively, in intra-bud morphogenesis compared with micro-patterning (Fig 4C). *LEF*, which encodes a transcription factor involved in the Wnt signaling pathway that participates in hair cell differentiation and follicle morphogenesis, was increased by 44-fold at E10 compared with E6 (Fig 4D). Surprisingly, several genes encoding negative regulators of the Wnt/β-catenin canonical signaling pathway, such as *DKK1*, *NKD1*, and *TCF1*, were consistently upregulated during intra-bud morphogenesis compared with micro-patterning (Fig 4E). Of these genes, *DKK1* was upregulated by 42-fold at E10 compared with E6–E8. DKK1might play an important role in the antagonism of the Wnt signaling pathway.

### The expression levels of genes related to the canonical Wnt/β-catenin signaling pathway changed significantly during feather follicle morphogenesis (E11–E14)

**Fig 5:**
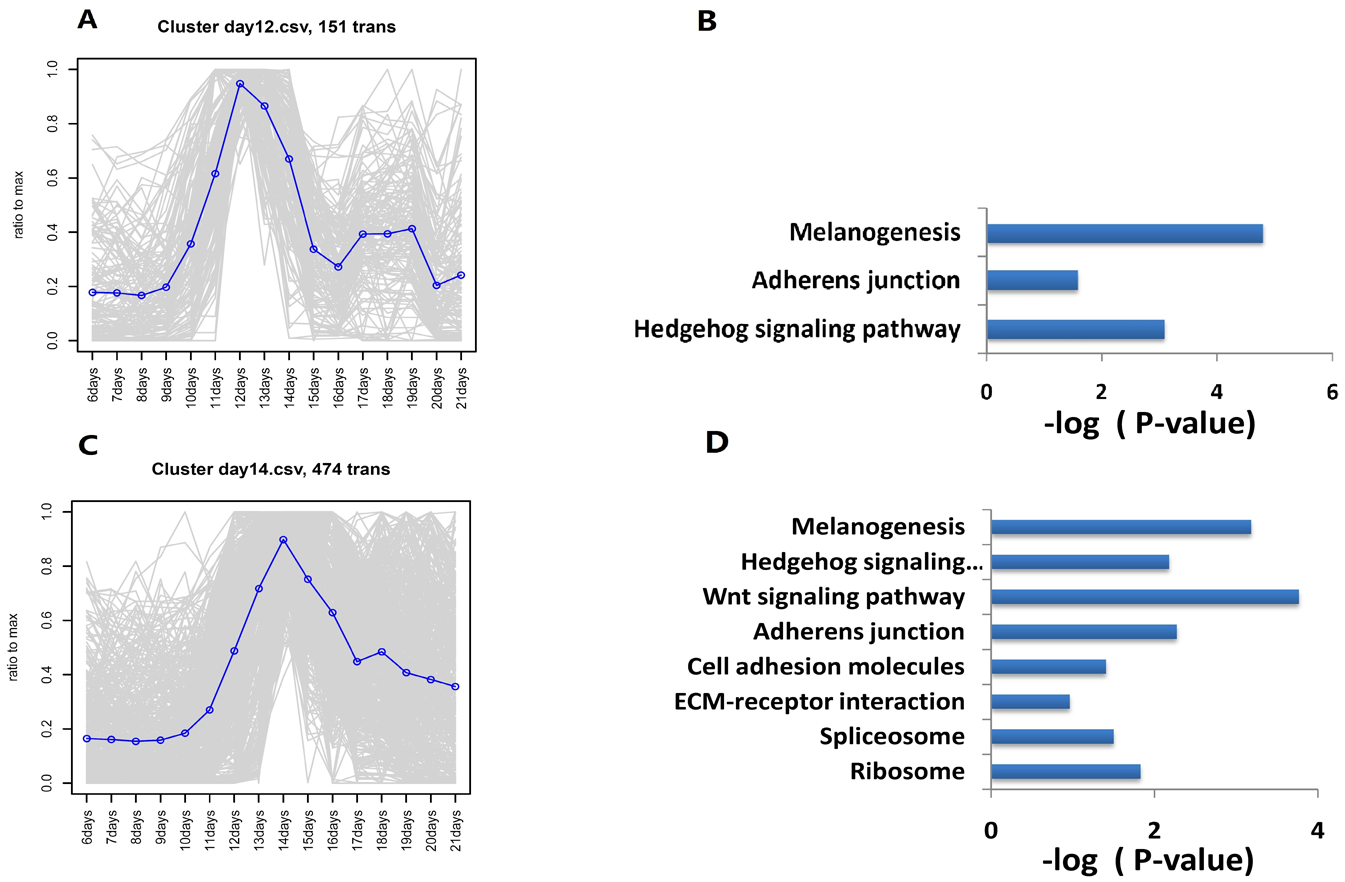
Representative time-course profile clusters in feather follicle morphogenesis and the results of pathway analysis. We clustered the profiles into coherent groups using weighted gene co-expression network analysis (WGCNA) and captured the two representative time-course profile clusters that included Cluster day12 (peaked at E12) (A), Cluster day14 (peaked at E14) (C), and performed Kyoto Encyclopedia of Genes and Genomes (KEGG) enrichment analysis for the set of genes in the two clusters (B, D).

The process of feather follicle morphogenesis involves temporal and spatial regulation of cellular processes, including localized cell proliferation, migration, adhesion, death, and differentiation. The histological results showed that the epidermis surrounding the base begins to invaginate into the dermis at E11. By E12 to E13, the fully-grown structure of the epidermis and dermis layer had developed and most feather primordia had become visible. A number of genes that were associated with feather follicle growth showed increased expression from E11 to E14. Pathway analysis identified several DEGs in Cluster day12 (peaked at E12) (Fig 5A) that were involved in melanogenesis (*CTNNB1*, *KIT*, *KITLG*, *TCF7*, *TYR*, *WNT16*, and *WNT5A*) and the hedgehog signaling pathway (*PTCH2*, *SHH*, *WNT16*, and *WNT5A*) (Fig 5B). By E14, the feather follicular cavities have disappeared because the feather germs completely fill their follicles. Pathway analysis showed that the DEGs in Cluster day14 (peaked at E14) (Fig 5C) are involved in the Wnt signaling pathway (*BAMBI*, *FZD6*, *SFRP2*, *TCF7*, *TP53*, *VANGL2*, *WNT10A*, *WNT16*, and *WNT6*), melanogenesis (*CREB3L1*, *FZD6*, *KITLG*, *TCF7*, *WNT10A*, *WNT16*, and *WNT6*), the hedgehog signaling pathway (*HHIP*, *WNT10A*, *WNT16*, and *WNT6*), and adherens junctions (*CDH1*, *EGFR*, *ERBB2*, *TCF7*, and *YES1*) (Fig 5D).

**Fig 6:**
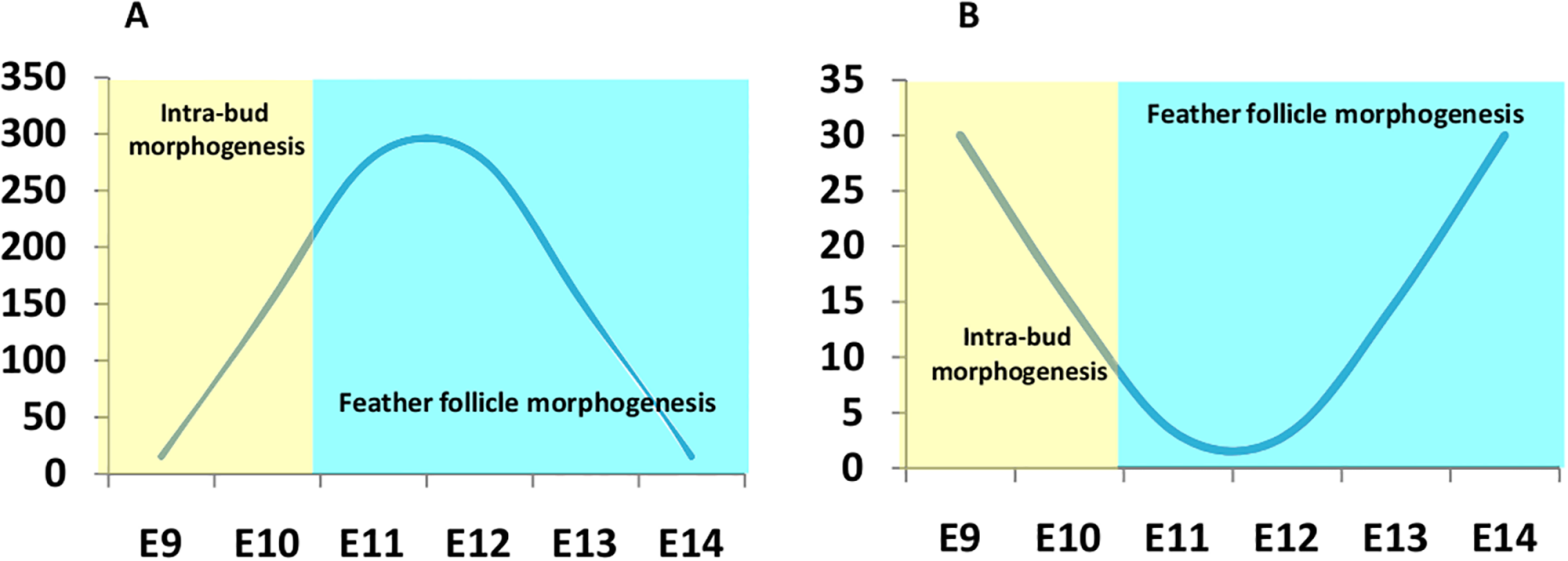
The expression trends of two Wnt signaling pathways in feather follicle morphology. (A) Schematic diagram of the expression of the canonical Wnt/β-catenin signaling pathway. (B) Schematic diagram of the expression of inhibitory factors of the canonical Wnt/β-catenin signaling pathway.

In this study, many DEGs were involved in the Wnt signaling pathway. Therefore, we decided to focus on the Wnt signaling cascade. The expression levels of genes encoding members of canonical Wnt/β-catenin signaling pathway increased. The transcription of genes encoding the ligands of the non-canonical Wnt pathway and the inhibitory factors of canonical Wnt/β-catenin signaling pathway were significantly decreased during feather follicle morphogenesis (Fig 6). We identified three Wnt signaling ligands, *WNT6*, *NT10A*, and *WNT16* that were upregulated by at least 7.1-,3.4-, and 28.6- fold from E10 to E14. The expression of *WNT11*, a gene encoding the ligand normally associated with the non-canonical Wnt/PCP (planar cell polarity) pathway, decreased during feather follicle morphogenesis. FZD6 is a Frizzled family receptor for the Wnt-protein ligand. *FZD6* was upregulated by 34.4-fold from E10 to E14, but downregulated from E6 to E10. *DKK1* and *NKD1* were downregulated during feather follicle morphogenesis. The expression of *RORA*, a gene encoding a nuclear receptor that inhibits canonical Wnt signaling by Wnt5a/PKC alpha-dependent phosphorylation [29], continued to increase after E12. *CTNNB1* and *LEF1* were downregulated from E14 (Table 2). These results suggested that the canonical Wnt/β-catenin signaling pathway is downregulated from E14 at the transcriptome level.

**Table 2:** Gene expression changes in Wnt signaling pathway in feather follicle morphogenesis.

Gene symbol	E10	E11	E12	E13	E14
**The ligands of canonical Wnt/ β-catenin signaling pathway**					
*WNT6*	1.9	1.1	6.4	12.7	13.6
*WNT10A*	3.8	5.6	10.4	12.4	13.1
*WNT16*	1.5	9.0	24.7	41.0	43.0
**The ligands of the non-canonical Wnt pathway**					
*WNT5A*	46.2	62.6	63.3	45.1	25.2
*WNT11*	20.9	17.9	11.1	8.6	7.8
**The membrane receptor of Wnt signaling pathway**					
*FZD6*	0.5	5.3	15.1	14.5	17.2
*FZD8*	0.6	0.4	0.3	0.4	0.9
*FZD10*	1.8	2.0	2.6	2.61	1.8
**The inhibitory factors of canonical Wnt/ β-catenin signaling pathway**					
*DKK1*	21.3	24.5	8.0	4.5	1.3
*DKK3*	0.7	1.0	0.2	0.2	0.04
*NKD1*	3.3	2.8	1.9	1.0	0.8
*RORA*	5.0	4.4	7.0	19.8	25.4
**The downstream component of the canonical Wnt signaling pathway**					
*CTNNB1*	144.3	293.4	290.7	152.9	2.8
*LEF1*	22.1	26.2	17.9	10.1	1.4

### Validation of the differential gene expression by qPCR

**Fig 7:**
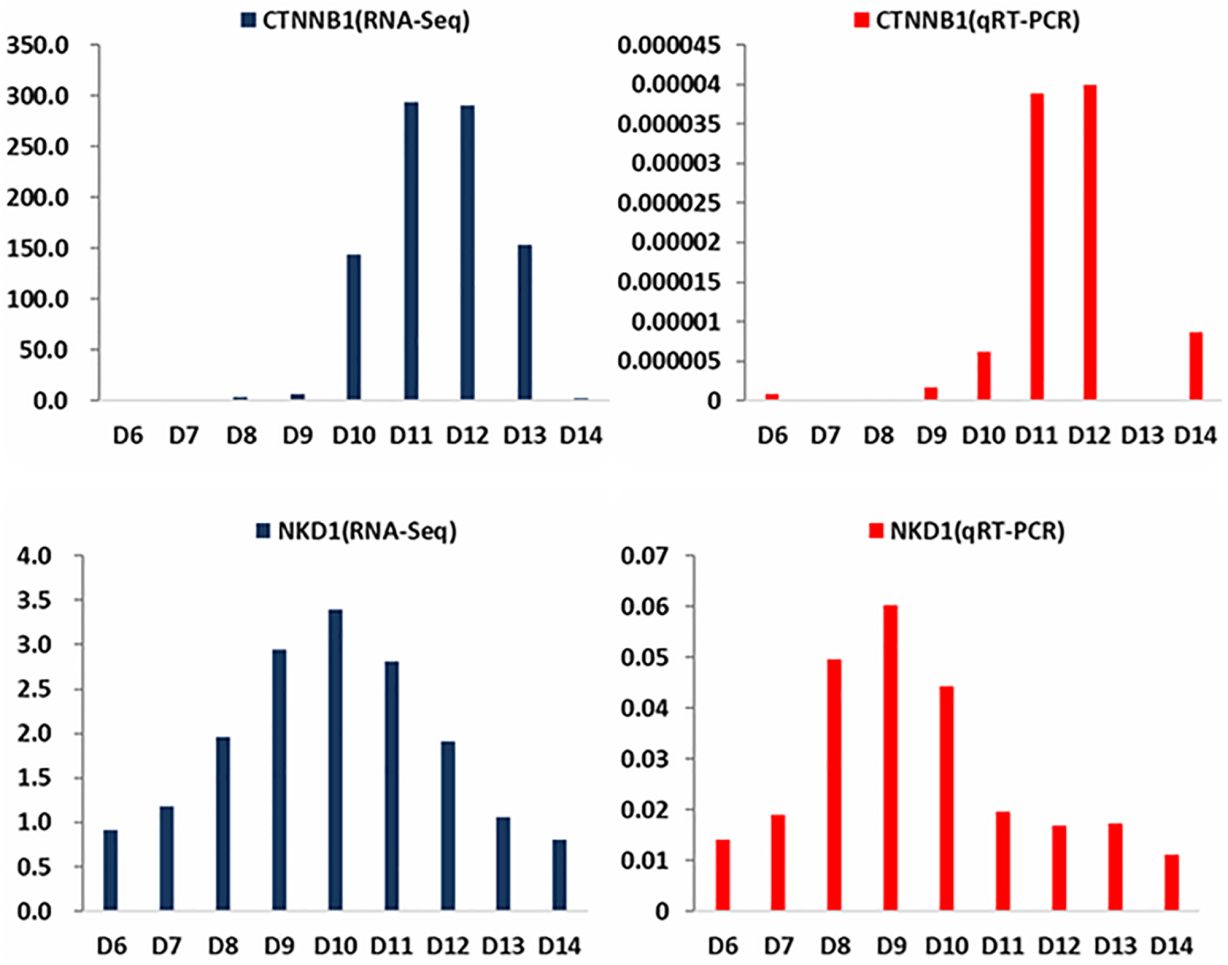
qPCR validation of gene expression including that of *CTNNB1* and *NKD1* from day6 to day14 of embryonic development.

Eleven genes were selected randomly for validation by qPCR. The results of *CTNNB1* and *NKD2* were shown in Fig 7, and the results for the other nine genes are shown in S3 File. Differences in expression values at some time points were found for several genes. However, only the expression of *CDC42* was inconsistent, which might be because the gene has a low expression level. However, the trends between the RNA-seq and qPCR analyses were the same for all 11 genes. The qPCR results verified that these 11 genes were differentially expressed at chick embryo skin during different development stages, which was consistent with the RNA-Seq data.

## Discussion

The time-course transcriptome analysis of the skin during chicken embryo development (E6 to E21) enabled us to interrogate the dynamic changes in global gene expression. Gene expression is the fundamental to phenotype[30]. Gene expression profiling has proved to be a popular method to distinguish developmental processes [31] and types of cancer [32]. Using this technique, we could investigate gene expression patterns in skin tissue at every developmental stage for every gene to recreate the entire developmental expression pattern. In this study, the result of hierarchical clustering of DEGs showed that the phase from E6–E21 could be divided into two large stages, feather follicle morphogenesis (E6–E14) and feather morphogenesis (E15–E21). The expression pattern of eigengenes can represent the different developmental stages. Traditionally, three stages have been defined in feather follicle morphogenesis (E6–E16). Our division had subtle differences from previous studies on morphology [5]. Feather follicle morphogenesis at the transcriptomic level was completed two days (E6–E14) in advance compared with the morphological level (E6–E16). Actually, sparse feathers were observed on the skin of the chicken embryos at E13. The feather germs already filled in the opening of the feather follicle by E14. After E14, the eigengenes related to the feather began to construct the structure of the feather. The key task switch of DEGs in the skin occurred at E14 from the morphology of the feather follicle to the construction of the feather. This dividing of developmental stages during chick feather morphogenesis could provide a new insight into the process of feather morphogenesis.

The module eigengenes related to non-canonical (Wnt/PCP (planar cell polarity)) and canonical Wnt signaling (Wnt/β-catenin) pathways showed dynamic changes during integument morphogenesis. The non-canonical pathway controls the orientation and differentiation of hair follicles of mice, which relates to skin patterning [33] and can polarize cells within the planar plane of an epithelium [34], playing a role during embryonic development; however, it is rarely mentioned during skin development [35, 36]. Results from KEGG analysis of the module genes suggested that *WNT5A*, *WNT11*, and *FZD6*, which encoded the ligands and membrane receptor protein of the non-canonical Wnt signaling pathway, were expressed at higher levels during intra-bud morphogenesis compared with that in the other periods. Wnt5A and Wnt11 could act cooperatively to attenuate canonical Wnt signaling [37] and inhibit the telogen-to-anagen transition of hair follicles [38, 39]. *FZD6* is the mediator of non-canonical pathways, and is required from E11.5 to E12.5 in mice embryos. The asymmetric arrangement of Merkel cells in each guard hair follicle depends on FZD6 expression in the epidermis [40]. In Fz6^-/-^ fetuses, the orientations of hair follicles in skin appear to be randomized [41], which implied that the Wnt/PCP signaling pathway was related to the initial skin axes development. In addition, *DKK1*, *NKD1*, and *TCF7* showed markedly higher expression inform that in E9–E10. DKK1 and NKD1 are antagonistic inhibitors of the canonical Wnt signaling pathway [42, 43]. *DKK1* also has an inhibitory effect on the differentiation of mesenchymal stem cells to fibroblasts [44–46].

**Fig 8:**
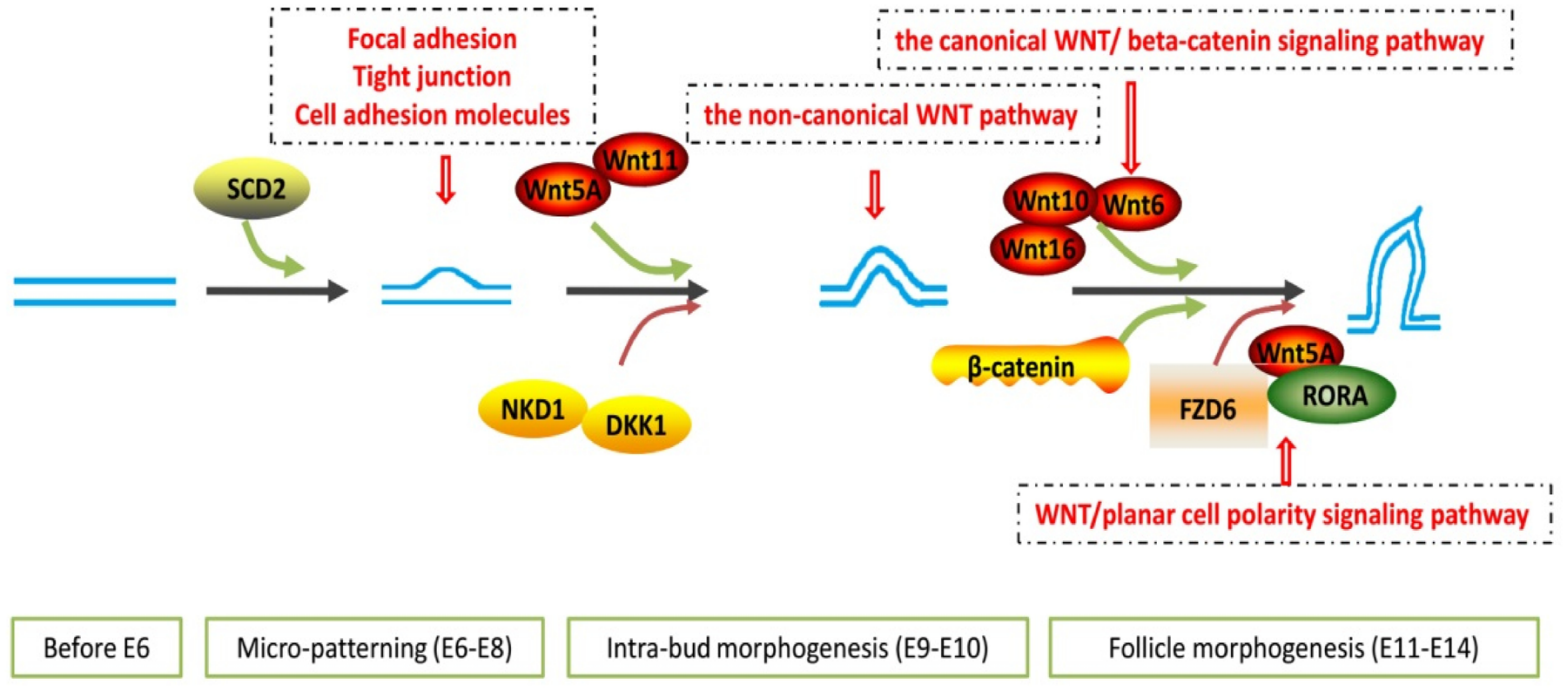
The dynamic regulation of the two kinds of Wnt pathways.

Wnt/β-catenin signaling is a central signaling pathway that regulates the embryonic integument morphogenesis [47] and adult hair follicle growth [48]. It is mediated by the intracellular molecule β-catenin (*CTNNB1*), which functions in both the regulation of cell-cell adhesion and in Wnt-dependent signal transduction [49]. In this study, *CTNNB1* was observed to be significantly upregulated during feather follicle morphogenesis (E11–E14). Research suggested that Wnt6, Wnt10, and Wnt16 are the ligands of the Wnt/β-catenin signaling pathway [50–52]. These genes showed similar trends of expression to *CTNNB1* during feather follicle morphogenesis. Wnt5A could activate β-catenin signaling in the presence of FZD4 [53–55] and suppress the canonical Wnt signaling pathway in the presence of ROR2 via a GSK3-independent pathway, which involves downregulation of β-catenin-induced reporter gene expression [56–59]. The results suggested that Wnt/β-catenin signaling at the transcriptome level was upregulated in the late stage of feather follicle morphogenesis. *RORA*, a gene encoding RAR Related Orphan Receptor A, was upregulated during the last two days of feather follicle morphogenesis. Thus, the Wnt5A/ROR pathway might be associated with activation of non-canonical Wnt signaling, and inhibition of canonical Wnt signaling during morphogenesis of feather follicles (E13–14). In addition, *FZD6* is upregulated during the late stage of feather follicle formation, which suggested that the FZD6-mediated Wnt/planar cell polarity signaling pathway has cross-talk with the Wnt/β-catenin signaling pathway in the late specification of feather follicle orientation (Fig 8). The two types of Wnt signaling pathways have different expression patterns in the polar development of skin cells during chicken embryonic development. The result suggested that the Wnt/β-catenin pathway may be downregulated by antagonistic inhibitors during intra-bud morphogenesis, and the non-canonical Wnt signaling might play an important role in establishing the direction of growth of the feathers by limiting epidermal choice.

In summary, our analysis on the time-course transcriptomes of skin morphogenesis during embryonic development revealed that genes and signaling pathways undergo dynamic changes in different skin morphogenesis stages. The expression patterns of DEGs showed that the feather follicle and feather morphogenesis phases could be subdivided during embryonic development of chickens, and this finding provides crucial insights into understanding the molecular process of feather morphogenesis. The results showed that those genes involved in two kinds of Wnt signaling pathway were differentially expressed in the skin during chicken embryo integument morphogenesis. This suggested that the Wnt pathway is under complex regulation and these genes can immediately switch on/off at different developmental stages. These results simply described the relation between the gene expression profiles of integument and skin morphogenesis during embryo development. In our future studies, we aim to perform functional analyses, such as in situ hybridization and immunostaining to determine the specific roles of the candidate genes in different developmental stages. We believe that this information will revive the interest of the research community in the fundamental process of feather morphogenesis.

## Supporting information

S1 File List of primers used in the qPCR analysis. (DOC)

S2 File Cluster analysis of 5830 differentially expressed genes by weighted gene co-expression network analysis (WGCNA). Twelve related clusters were generated. Each cluster is named according to the number of days (highest peak), and includes the number of transcripts at the end.
(PDF)

S3 File qPCR validation of differentially expressed genes from day6 to day14 of chicken embryo development. (DOC)

S4 File NC3Rs ARRIVE guidelines checklist. (DOCX)
