# Supplementary material for: Skin transcriptome reveals the dynamic changes in the Wnt pathway during integument morphogenesis of chick embryos
Source: PLoS One. 2018 Jan 19;13(1):e0190933. doi: 10.1371/journal.pone.0190933 (PMC5774689; doi:10.1371/journal.pone.0190933)
Supplement: S1 File — (DOC) [file pone.0190933.s001.doc]

| **Gene** | Tm℃ | Forward primer(5'-3') | Reverse primer(5'-3') |
| --- | --- | --- | --- |
| ***LEF1*** | 62 | ATCCAACAAGGTGCCAGTG | GGCTGCCTGAATCCACTC |
| ***WNT5A*** | 58 | ACCTCGTAGTGGCTCTGG | AACTGATACTGGCATTCCTTA |
| ***COL2A1*** | 66 | TGGAGCGCAGATGGGTGTC | GTAGCCTCGGTGGCCTTTCA |
| ***COL9A1*** | 64 | GGTCCCATTGGTGAAGTTG | GGACCCTCTTCTCCCACT |
| ***COL9A2*** | 64 | TTGACGTGGTGCTGAAGATGAT | CCTTTGGGTCCGATGTTGC |
| ***COL9A3*** | 58 | CACTCTTGGGCTACTCTTC | TTCCTGTTAAACCGTCTACT |
| ***NKD1*** | 58 | GACAACAGCACCTGACCC | GCAAACTCCATCCGTCTT |
| ***CTNNB1*** | 64 | TTGTTCTATGCCATTACTACTC | ACACCTTCAGCACCCTAC |
| ***WNT10A*** | 64 | CAGGGACATCCACTCACGC | TGCCAGCAGGTCTTCAGC |
| ***CDC42*** | 58 | GGGACCTGAAGGCTGTT | GGTGGCTTTATTCGTTTT |
| ***WNT16*** | 58 | TGCTCCTGTGACACCAAACT | CACGACCCAGAAACACCAT |

Suppl. Table1: primer information of qRT-PCR validation
