# Supplementary material for: Skin transcriptome reveals the dynamic changes in the Wnt pathway during integument morphogenesis of chick embryos
Source: PLoS One. 2018 Jan 19;13(1):e0190933. doi: 10.1371/journal.pone.0190933 (PMC5774689; doi:10.1371/journal.pone.0190933)
Supplement: S2 File — Twelve related clusters were generated. Each cluster is named according to the number of days (highest peak), and includes the number of transcripts at the end. (PDF) [file pone.0190933.s002.pdf]

Cluster day6.csv, 717 trans

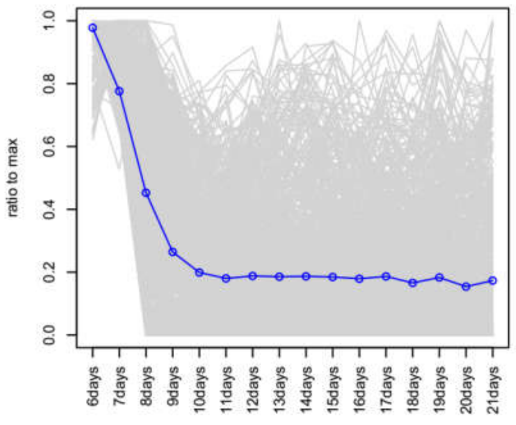

Cluster day6+12.csv, 570 trans

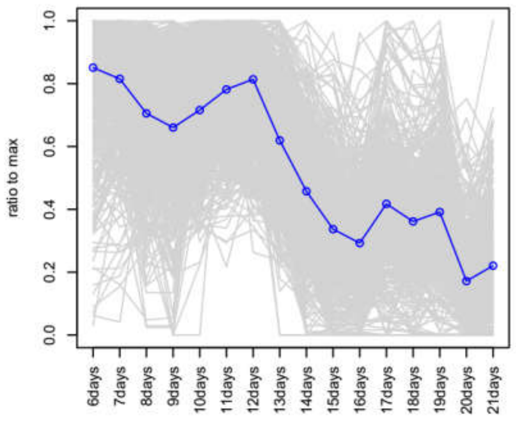

Cluster day8.csv, 417 trans

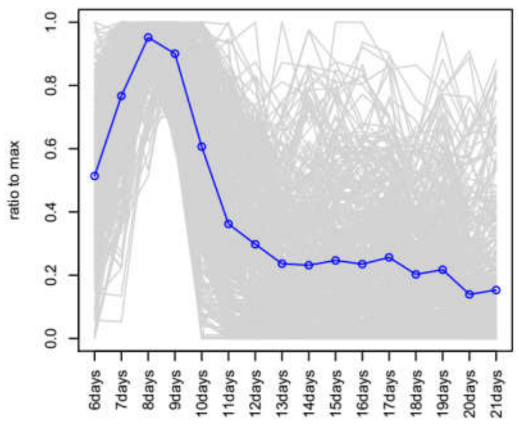

Cluster day8+16.csv, 44 trans

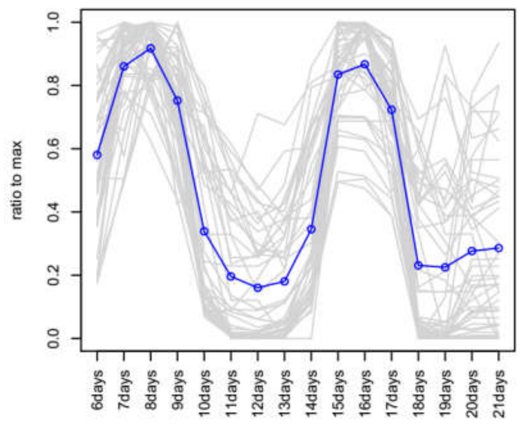

Cluster day10.csv, 173 trans

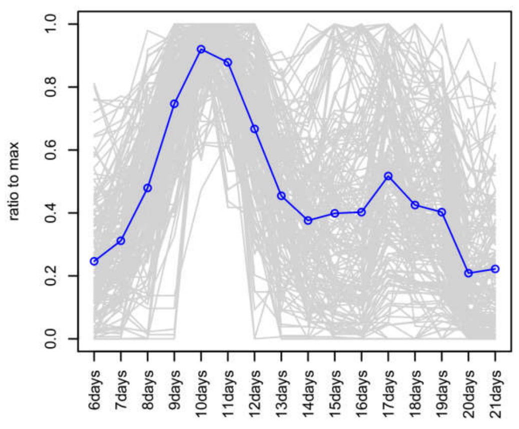

Cluster day12.csv, 151 trans

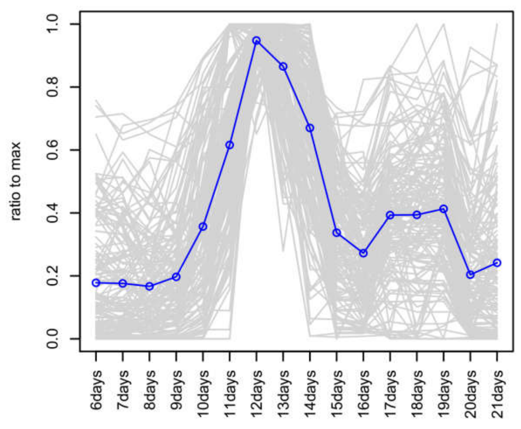

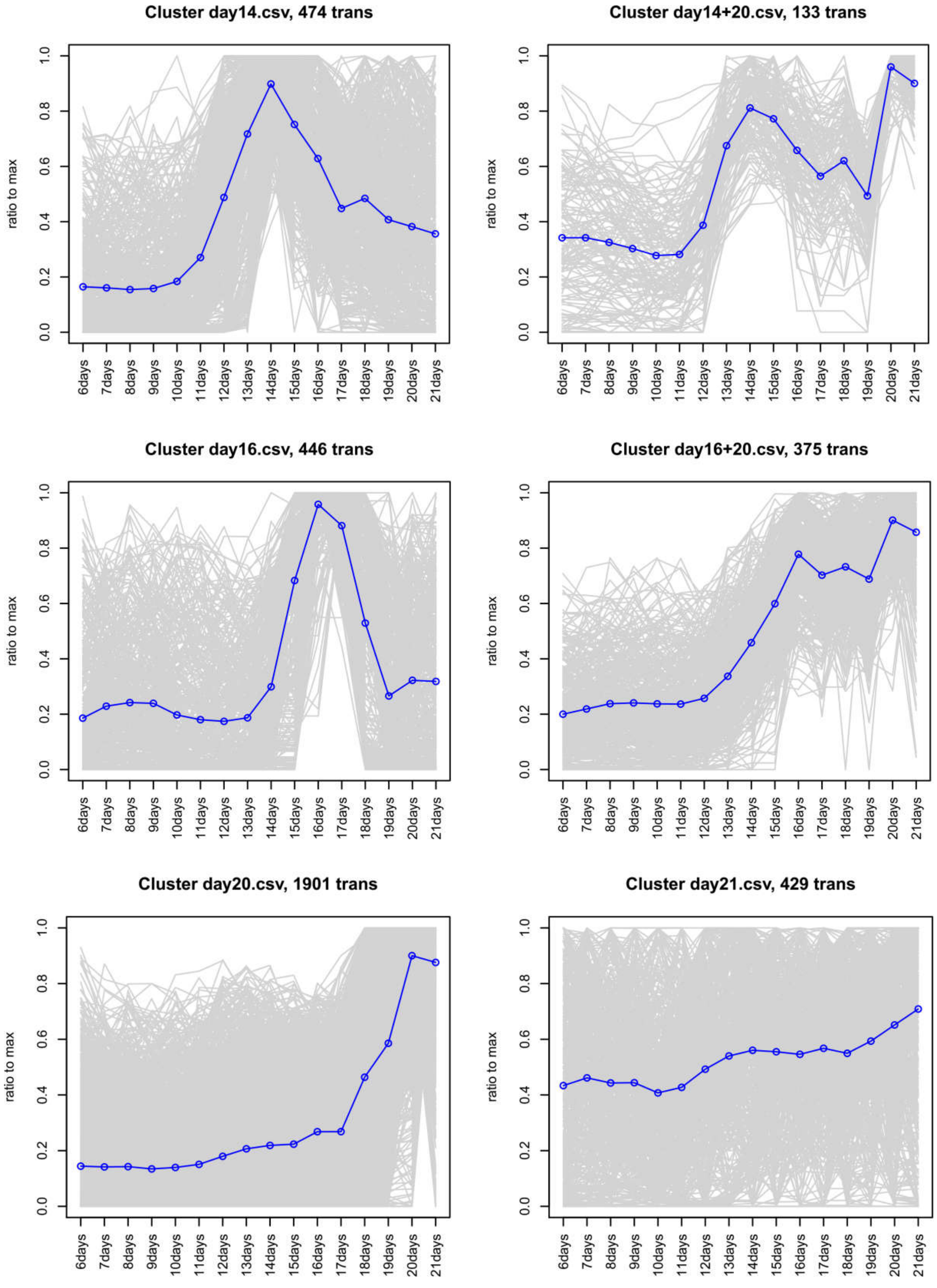

Suppl.Figure 2: Cluster analysis of 5830 differentially expressed genes by WGCNA
