# Supplementary material for: Skin transcriptome reveals the dynamic changes in the Wnt pathway during integument morphogenesis of chick embryos
Source: PLoS One. 2018 Jan 19;13(1):e0190933. doi: 10.1371/journal.pone.0190933 (PMC5774689; doi:10.1371/journal.pone.0190933)
Supplement: S3 File — (DOC) [file pone.0190933.s003.doc]

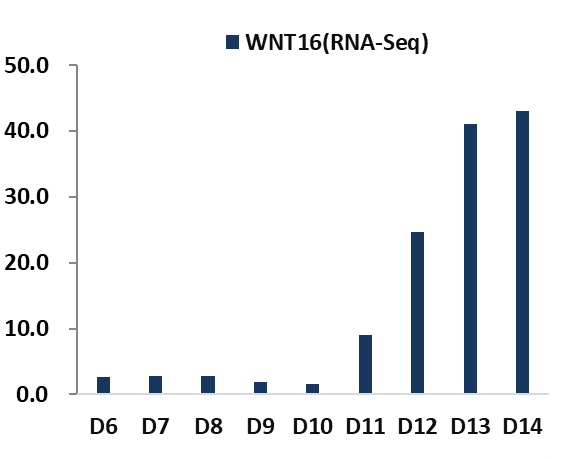

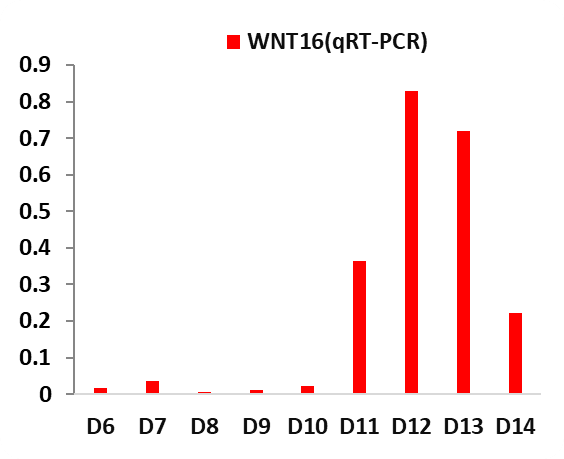


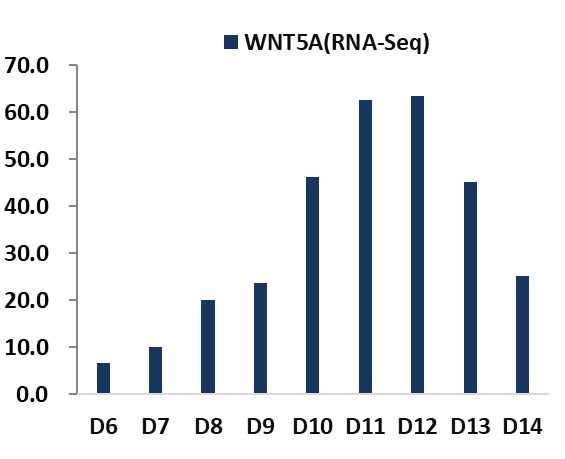

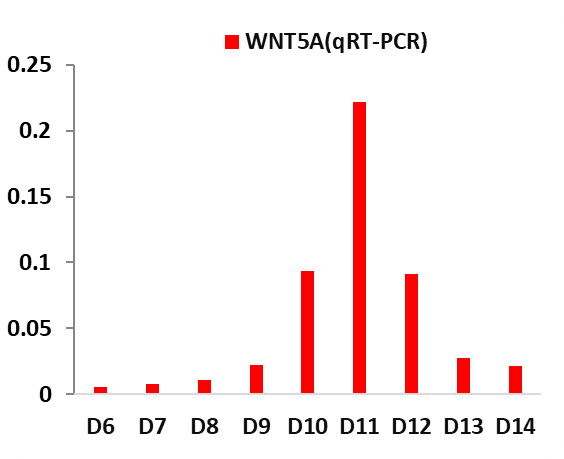


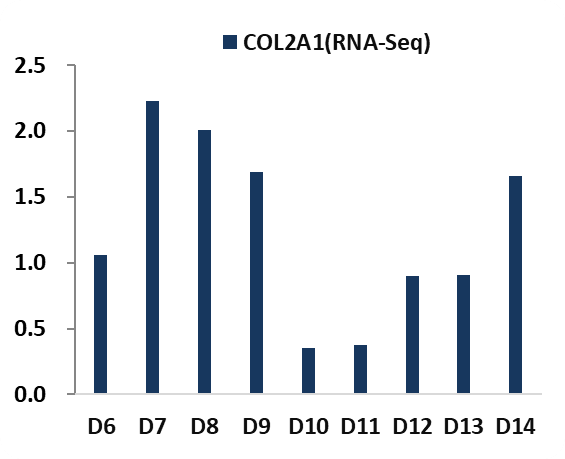

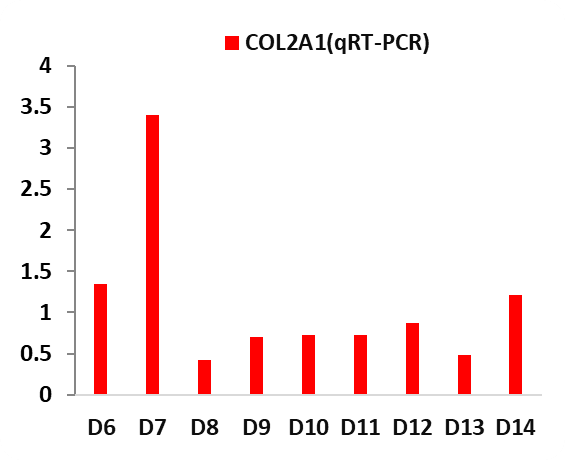


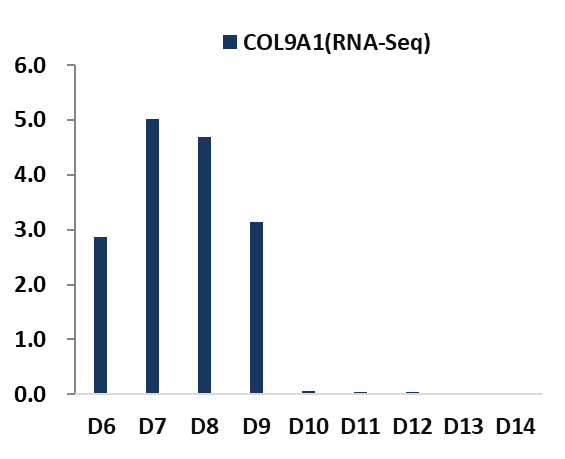

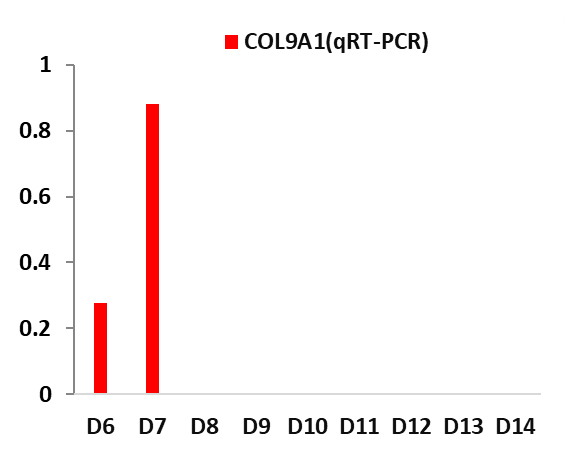


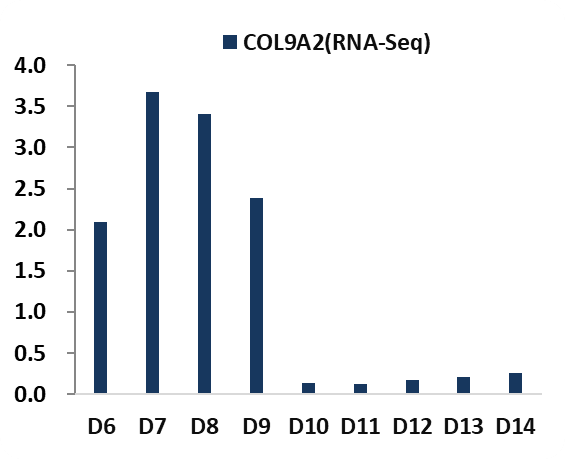

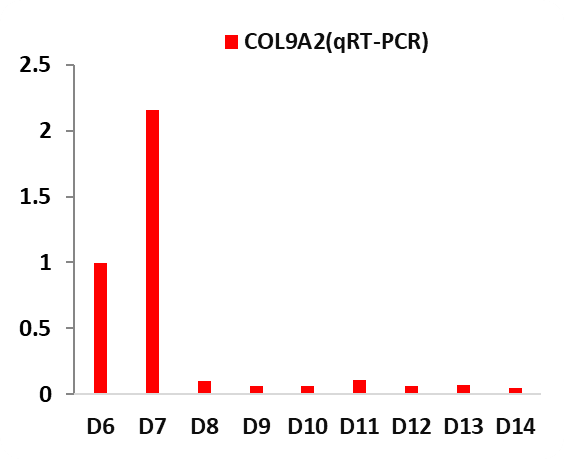


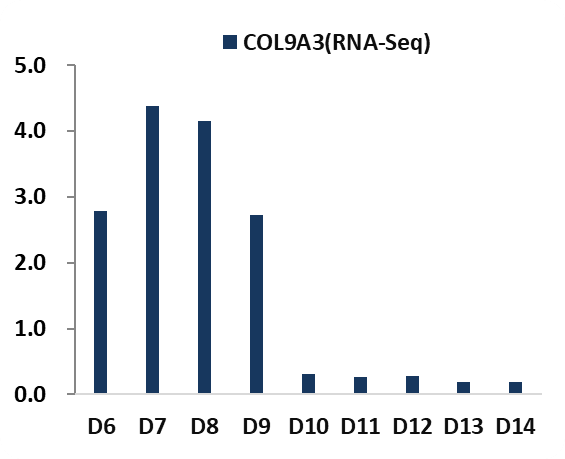

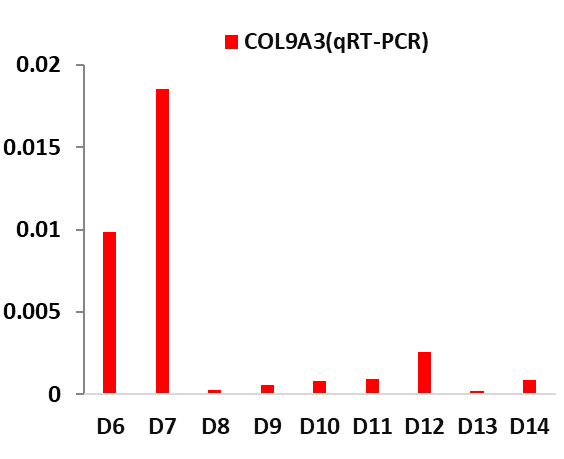


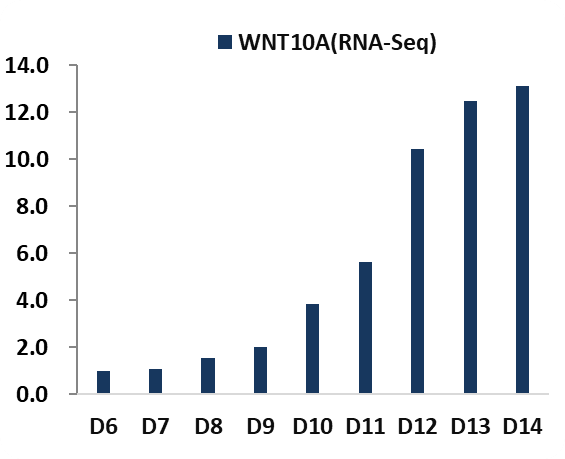

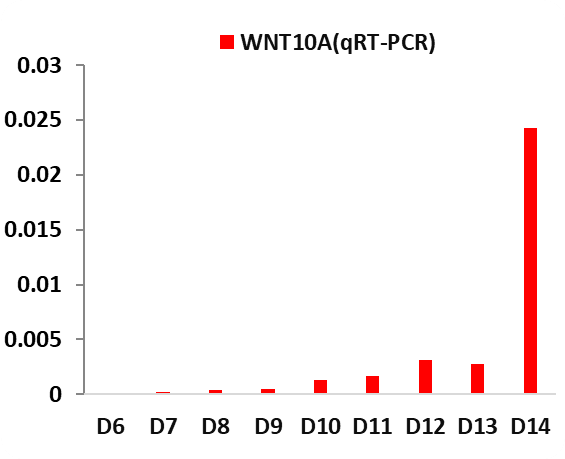


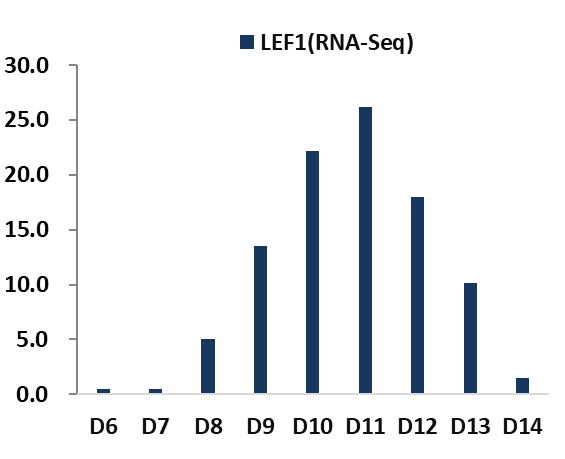

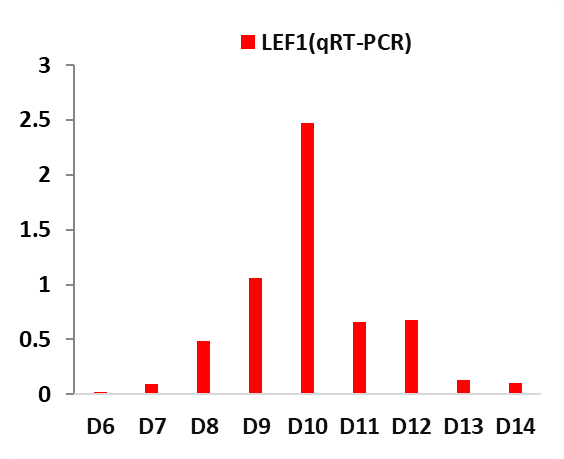


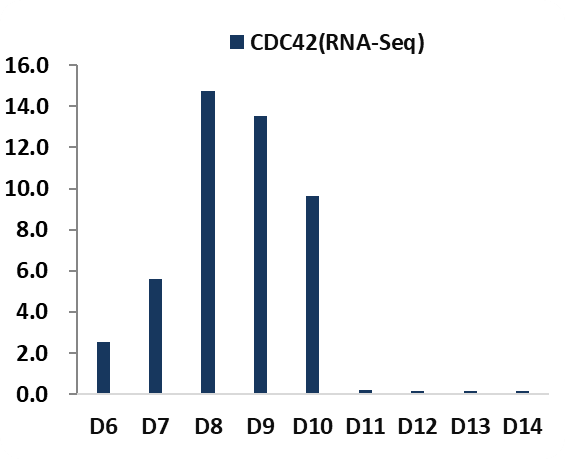

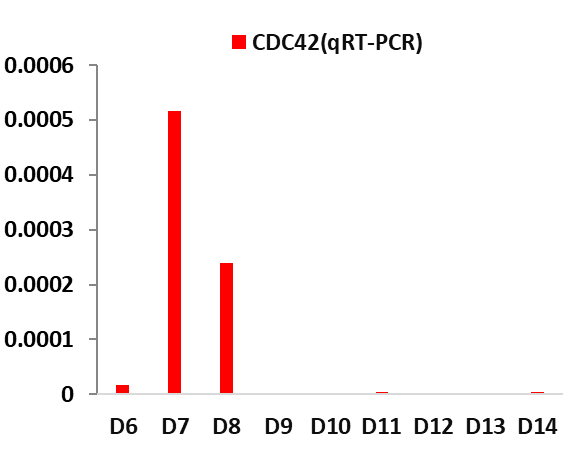


Suppl. Figure2: qRT-PCR validation ofdifferent expressed genesduring embryonic development day6 to day14
