# Supplementary material for: Skin transcriptome reveals the dynamic changes in the Wnt pathway during integument morphogenesis of chick embryos
Source: PLoS One. 2018 Jan 19;13(1):e0190933. doi: 10.1371/journal.pone.0190933 (PMC5774689; doi:10.1371/journal.pone.0190933)

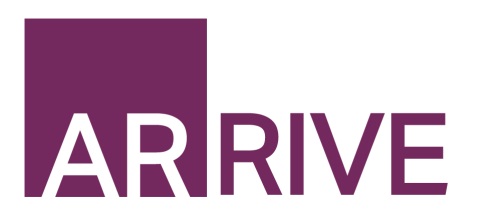


The ARRIVE Guidelines Checklist

Animal Research: Reporting In Vivo Experiments

Carol Kilkenny^1^, William J Browne^2^, Innes C Cuthill^3^, Michael Emerson^4^ and Douglas G Altman^5^

*^1^The National Centre for the Replacement, Refinement and Reduction of Animals in Research, London, UK, ^2^School of Veterinary Science, University of Bristol, Bristol, UK, ^3^School of Biological Sciences, University of Bristol, Bristol, UK, ^4^National Heart and Lung Institute, Imperial College London, UK, ^5^Centre for Statistics in Medicine, University of Oxford, Oxford, UK.*

|  | ITEM | RECOMMENDATION | Section/ Paragraph |
| --- | --- | --- | --- |
| 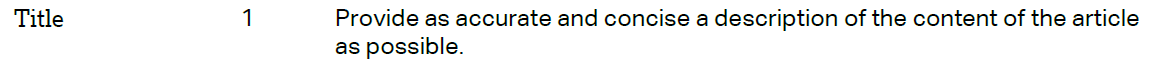 | | | Title |
| 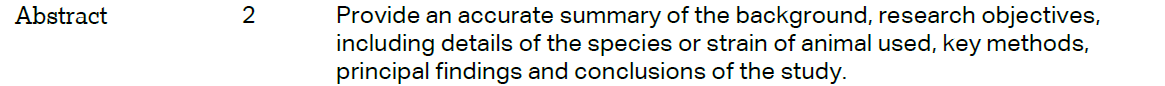 | | | Abstract |
| INTRODUCTION | | |  |
| 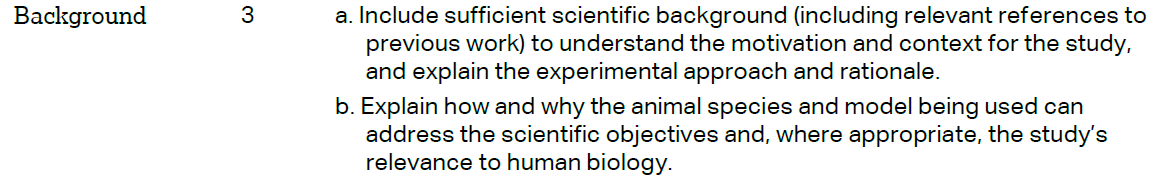 | | | Paragraphs  1-4  Paragraphs  3-4 |
| 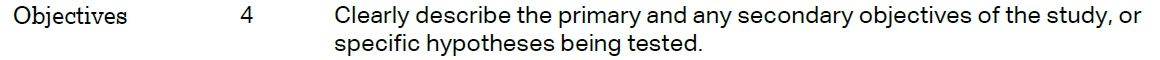 | | | Paragraphs4 |
| METHODS | | |  |
| 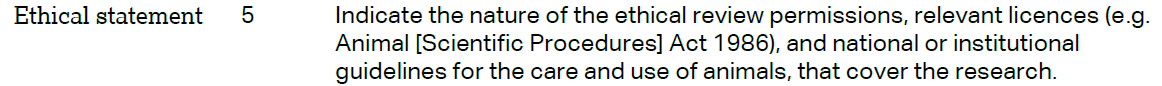 | | | Paragraphs5 |
| 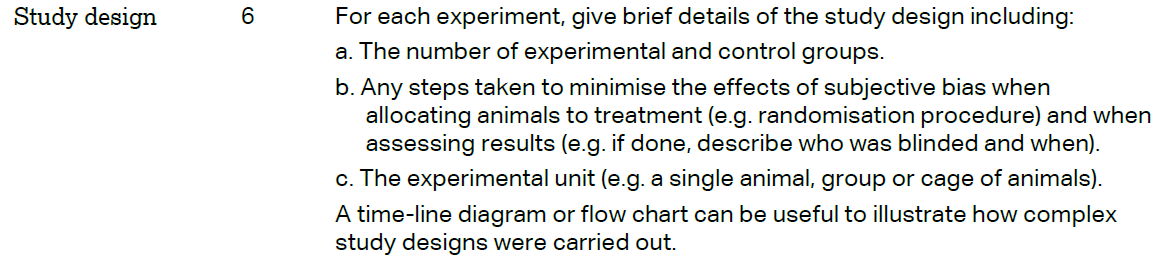 | | | Paragraphs6-13 |
| 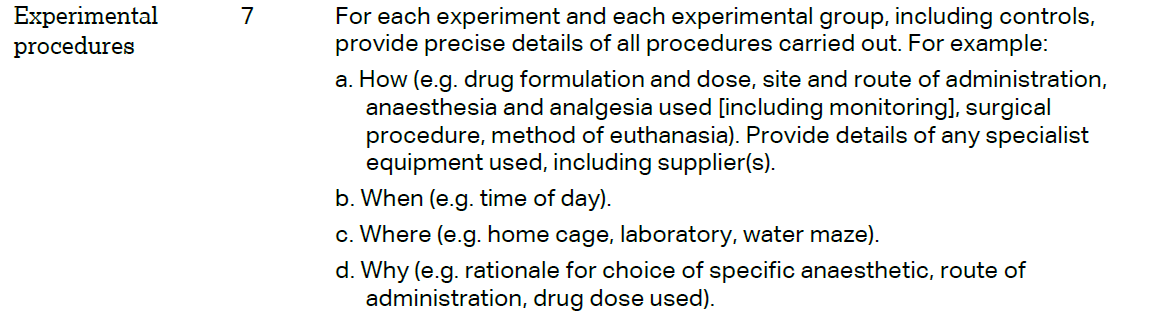 | | | Paragraphs6-13 |
| 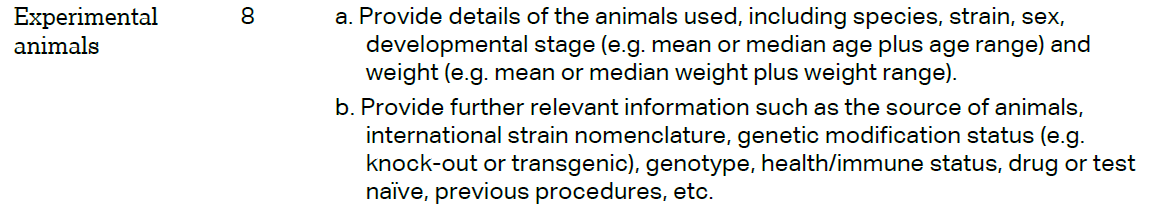 | | | Paragraphs6 |

The ARRIVE guidelines. Originally published in *PLoS Biology*, June 2010^1^

| 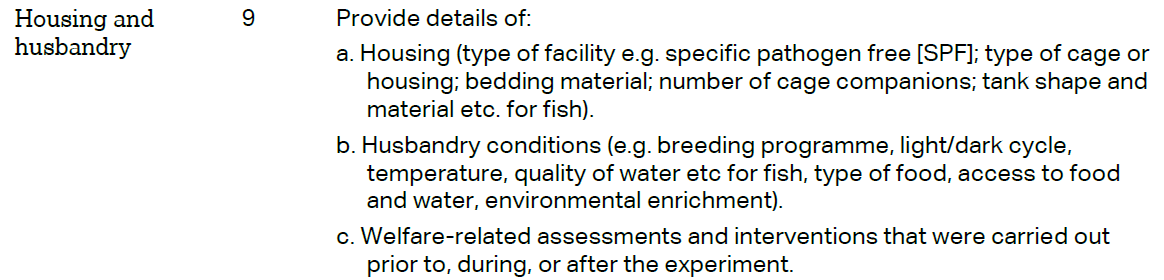 | Paragraphs6 | |
| --- | --- | --- |
| 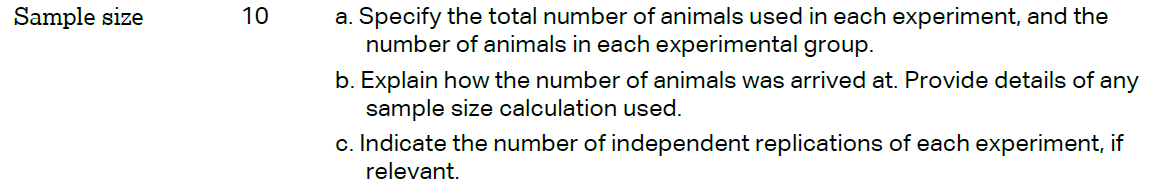 | Paragraphs7 | |
| 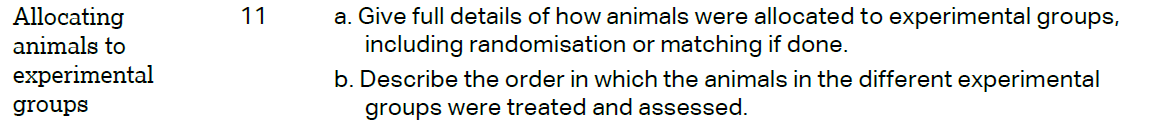 | Paragraphs5 | |
| 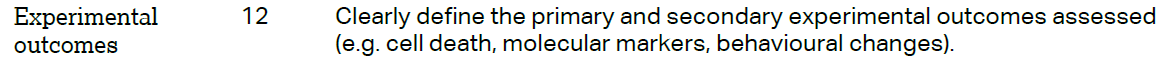 | Paragraphs7-12 | |
| 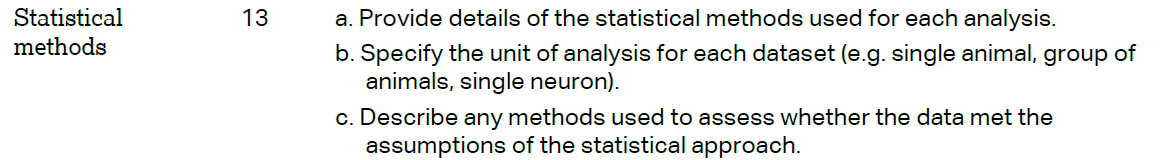 | Paragraphs8-10,13 | |
| RESULTS |  | |
| 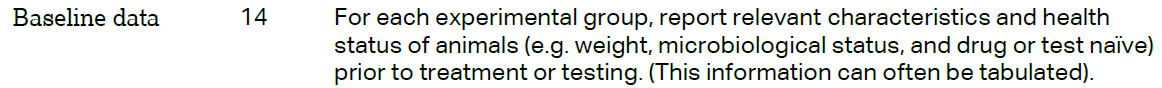 | Paragraphs14-26 | |
| 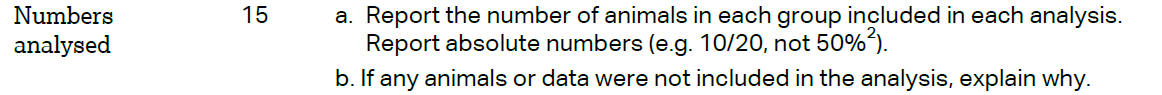 | Paragraphs14-26 | |
| 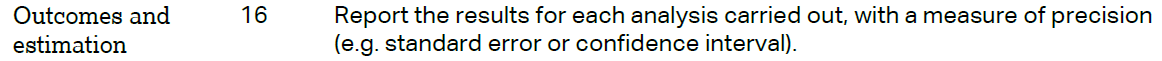 | Paragraphs14-26 | |
| 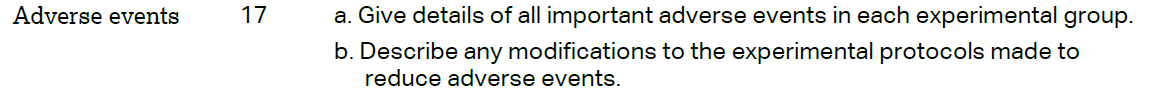 | Paragraphs14-26 | |
| DISCUSSION |  | |
| 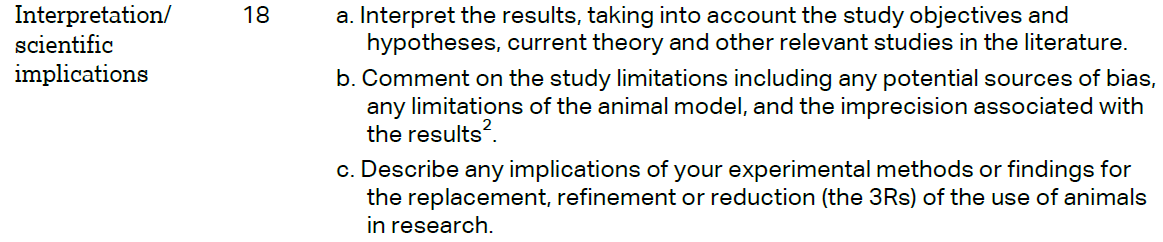 | Paragraphs127-30 | |
| 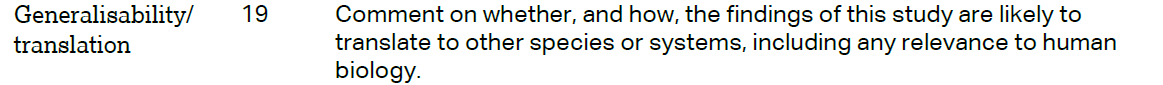 | Paragraphs128-30 | |
| 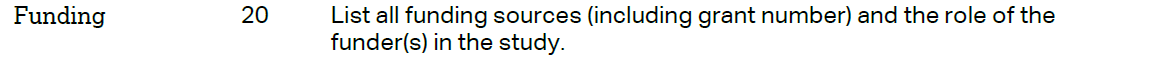 |  |  |


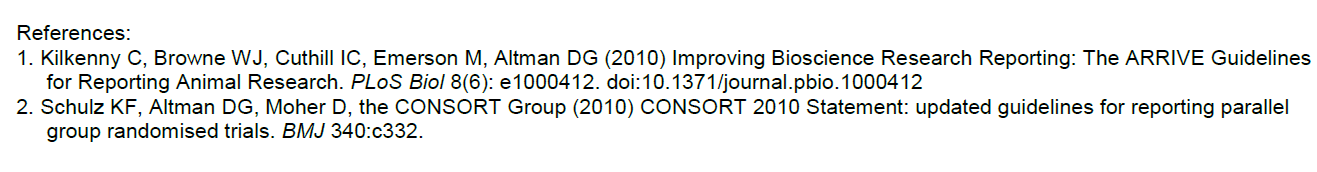

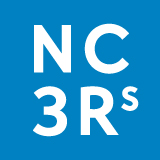

Supplement: S4 File — (DOCX) [file pone.0190933.s004.docx]
